# A7 CARBOXYMETHYLCELLULOSE SENSITIVE GUT MICROBIOME SIGNATURES AND THEIR IMMUNO-METABOLOMIC LINKS TO CROHN’S DISEASE

**DOI:** 10.1093/jcag/gwaf042.007

**Published:** 2026-02-13

**Authors:** J Kim, H Rytter, Q Li, C McShane, S Jeong, H Steinhart, S Lee, K Croitoru, B Chassaing, W Turpin

**Affiliations:** University of Toronto, Toronto, ON, Canada; Microbiome-Host Interactions, Institut Pasteur, Université Paris Cité, INSERM U1306, Paris, France; University of Toronto, Toronto, ON, Canada; University of Toronto, Toronto, ON, Canada; University of Toronto, Toronto, ON, Canada; University of Toronto, Toronto, ON, Canada; University of Toronto, Toronto, ON, Canada; University of Toronto, Toronto, ON, Canada; Microbiome-Host Interactions, Institut Pasteur, Université Paris Cité, INSERM U1306, Paris, France; University of Toronto, Toronto, ON, Canada

## Abstract

**Background:**

A recent study reported that carboxymethylcellulose (CMC), a common synthetic emulsifier, can disrupt the gut microbiota, potentially triggering gut inflammation and metabolic disturbances. A follow up study revealed interindividual variability in CMC effects liked to microbiota CMC sensitivity, identifying 78 associated metagenomic markers.

**Aims:**

We aimed here to evaluate whether these 78 CMC-sensitive metagenomic markers were associated with risk of development of Crohn disease (CD), and to examine how ultra-processed food (UPF) consumption might impact these associations.

**Methods:**

The CCC-GEM project prospectively enrolled healthy first-degree relatives (FDRs) of CD patients. Deep shotgun metagenomic sequencing, serum metabolomics, and serology were analyzed in a subset of GEM cohort. UPF consumption was classified using the NOVA system, with high UPF intake defined as ≥ 40% of total energy. Associations between metagenomic signatures and CD development and their relationships with metabolomic and serologic profiles, were evaluated using Cox proportional hazards and generalized estimating equation models. Statistical significance was defined at a false discovery rate-adjusted q value<0.05.

**Results:**

A total of 854 healthy FDRs were assessed, including 72 who later developed CD and 782 who remained healthy during a median follow-up of 9.2 years. Seven of the 78 metagenomic signatures (A0A3E5GJN6, A0A174HK87, R7AI59, R6SSX3, R7AZQ0, UPI000E4942B7, R7A8T1) were significantly associated with an increased risk of CD development. Continuous interaction analysis revealed that high UPF consumption potentiated the risk conferred by the marker A0A2Y4XX73 (HR per 10% increase 2.48, 95% CI 1.45-4.26, q = 0.064). Among individuals with high UPF consumption, A0A2Y4XX73 showed a trend toward an increased risk of CD (Hazard ratio [HR] 3.88; 95% confidence interval [CI] 1.66-9.06; q = 0.130, whereas no significant association was observed in the low UPF consumption group (HR 0.74; 95% CI 0.28-1.99; q = 0.859). This metagenomic signature was also associated with increased anti-flagellin IgA antibody responses (VAR_14_2Fla, A4Fla4, and CBir66, q < 0.049) and with an increase of a serum biomarker of oxidative stress (methionine sulfone) (q = 1.3 × 10^-5^).

**Conclusions:**

Our findings identified select CMC-sensitive microbial functions associated with CD risk. Importantly, select metagenomic markers appear to have a strong association with CD risk among individuals with high UPF consumption, suggesting that dietary exposure to UPFs may amplify the pathogenic potential of CMC-sensitive microbiome profiles. Furthermore, the observed associations between these microbial functions and immune responses to flagellin and oxidative stress highlight a potential mechanistic link in CD pathogenies.

**Funding Agencies:**

CCC, CIHRHelmsley Charitable trust

